# The Mental Health Outcomes of Food Insecurity and Insufficiency in West Africa: A Systematic Narrative Review

**DOI:** 10.3390/bs11110146

**Published:** 2021-10-25

**Authors:** Kenneth Ayuurebobi Ae-Ngibise, Winifred Asare-Doku, Jennifer Peprah, Mohammed Nuhu Mujtaba, Diane Nifasha, Gordon Maanianu Donnir

**Affiliations:** 1 School of Medicine and Public Health, College of Health, Medicine and Wellbeing, The University of Newcastle, Callaghan, NSW 2308, Australia; jennifer.peprah@uon.edu.au (J.P.); gordon.donnir@uon.edu.au (G.M.D.); 2 Kintampo Health Research Centre, Kintampo P.O. Box 200, Bono East Region, Ghana; mmujtaba24@yahoo.com; 3 National Drug and Alcohol Research Centre, Faculty of Medicine, University of New South Wales, Sydney, NSW 2052, Australia; Winifred.asaredoku@uon.edu.au; 4 Positive Mental Health Program, 53 Willandra Dr., Epping, VIC 3076, Australia; dnifasha@gmail.com; 5 Department of Psychiatry, Komfo Anokye Teaching Hospital, Kumasi P.O. Box 1934, Ashanti Region, Ghana

**Keywords:** famine, food insufficiency, food insecurity, mental health, cognitive development, West Africa

## Abstract

(1) Background: Food insufficiency is a global pandemic affecting many people, especially those residing in developing countries. African countries have been affected by food insufficiency, which is mostly caused by drought or wars. Famine or food insufficiency has been reported to have an impact on the psychological health and quality of life of people affected. This review assessed the mental health outcomes of famine and food insufficiency in West Africa. (2) Methods: A search of the published literature was conducted using PubMed, PsycExtra, Medline, and PsycINFO databases. The search was limited to papers published in English between the years 2010 and 2020. Two reviewers independently screened the titles and abstracts of the retrieved papers using pre-defined inclusion and exclusion criteria and a third reviewer resolved conflicts. Data were extracted and appraised using a data extraction form and an appraisal checklist. (3) Results: A total of 81 papers were identified through the journal databases search. Out of the seven papers that met the inclusion criteria, six papers used cross-sectional designs and one paper used an experimental design. The six papers used quantitative approach for data collection, while the one paper used a qualitative technique. The evidence synthesized from this review indicated that exposure to food insecurity or insufficiency is associated with increased psychological distress including anxiety, sleeplessness, intellectual disability, general mental, and emotional instability. (4) Conclusions: This review strongly highlights the need for further research across the sub-region. It further suggests that famine and food insufficiency are associated with significant mental health problems in adults and impacts the cognitive and intellectual development of children. Although there is paucity of literature about famine and its impact on mental health in West Africa, these findings are important for developing social policy initiatives for increasing food supply and mental health interventions for all ages.

## 1. Introduction

The United Nations Food and Agriculture Organization (FAO) indicates that 925 million people in the world are suffering from hunger with many undernourished people living in Asia, the Pacific Islands, and Africa [1]. According to the United Nations, hunger can be described as periods when the population is facing severe food insecurities in which people can go without food for days, due to lack of money, food access, and/or other resources [1].

Food security is a complex issue and defined as “a state where all people, at all times, have physical and economic access to sufficient, safe and nutritious food to meet their dietary and food preferences for an active and healthy life” [2]. Food insecurity has been described as the limited or uncertain availability of nutritionally adequate and safe foods or limited or uncertain ability to acquire acceptable foods in socially acceptable ways; whereas food insufficiency has been described as an extreme form of household food insecurity where household members sometimes, or often, do not have enough to eat [3,4]. African countries have been affected by food insecurity and, according to the statistics, 98 million have faced food shortage in 2020 alone [5]. Famine or food insecurity are mostly caused by conflict, climate variability and extremes, inefficient food supply chains, and economic downturns in Africa [1]. Despite growing interest in the mutually reinforcing association between poverty and mental ill-health, there is lack of published evidence in Africa [6].

According to the FAO, Sub-Saharan Africa (SSA) is the most affected by extreme poverty; one in four experience chronic poverty. For individuals within these countries 40–50% live below the average earnings of $1.25, making SSA and Southern Asian countries one of the world’s poorest regions. Due to the extreme poverty, many governments lack the support systems to help those who are finding it hard to feed their families [7]. In 2011/12, East Africa experienced the worst famine of the past 25 years. This was during the war in Somalia where 260,000 people suffered the worst starvation to death, this included 133,000 children under the age of five [7].

West Africa has experienced its fair share of drought and famine [8,9]. The West African region is important to focus on because, the Economic Community of West African States (ECOWAS) supported by the United Nation’s FAO initiated a Zero Hunger Initiative in 2014 to improve food security and nutrition in the sub-region [10], in consonance with the Millennium Development Goal 1 which targets the eradication of extreme poverty and hunger globally. This was in line with the new target of 2025 set by the World Health Organisation (WHO) to improve global nutrition and diet of malnourished children and adolescents [11]. Although there has been some 63 percent reduction in the proportion of West Africans suffering from hunger, this reduction is insufficient, and the sub-region is lagging behind according to global trends [10]. In addition, due to political and civil security instability posed by insurgent groups such as Boko Haram and ISIS-West Africa in Nigeria and other countries, there have been massive internal displacements resulting in food and nutrition insecurity [12]. Furthermore, climate change and reduction in rainfall in West Africa has posed significant threat to agriculture and food security [13,14]. 

Studies have identified that long term hunger can have both physical and psychological effects on individuals [15]. Food insufficiency has impacts on the mental health of individuals with poorer mental health outcomes among children in particular [16,17]. There are also reports that suggest women are disproportionately impacted by food insufficiency in low- and middle-income countries. A study in South Africa among women found that most households were chronically food insecure leading to worries and stress about food [18]. Similar findings have been reported among Ghanaian women [19], in Thailand [20], and among Women in Botswana and Swaziland where food insufficiency is an important risk factor for increased sexual risk-taking [21]. Some earlier research indicates that food insecurity may be a stronger predictor of poor mental health outcomes compared to other forms of insecurity, due to the important biological and social function of food in the lives of people [22]. Global evidence suggests that food insecurity is linked with poorer mental health outcomes; for instance, in New Zealand a strong relationship was found between food insecurity and psychological distress [23]. Similar findings have been reported in the United States [24], Bangladesh [25], Ghana [26], and Canada [27]. Hadley, et al. [28] have suggested that there is likely to be a positive influence on adult mental health with interventions that aid food security due to the high association between these variables.

There is severe dearth of published literature about the impact of food insecurity and famine on mental health outcomes in West Africa, as the majority of existing evidence is from developed countries. Undoubtedly, there is an indication for more evidence to unveil the potential impact of famine and food insufficiency on mental health in this region. Understanding the extent and effect of food insufficiency on mental health outcomes can inform relevant policy [-ies] and interventional implications for stakeholders, including governments and donor organizations, about the need to invest resources in food production which will subsequently mitigate the impact of mental ill-health. This review seeks to highlight the gap in the literature, and the need to prioritize research focusing on food insecurity and mental health in West Africa by systematically pulling together the existing evidence from West Africa on the mental health impact of famine and food insufficiency.

## 2. Materials and Methods

### 2.1. Search Strategy

Two authors searched Pubmed, PsycExtra, Medline, and PsycINFO databases. The search was conducted using the following terms: search term group 1—“mental health”, “post-traumatic stress disorder”, “anxiety disorders”, “mood disorders”, “stress disorders”, and “cognitive development”; search term group 2—“food insufficiency”, “famine”, “food shortage”, hunger”, “starvation”, and “food insecurity”. The search terms were combined with “OR” and “AND”, including articles from 2010 to May 2020. This timeframe was chosen to examine new evidence since the development of the Millennium Development Goals. A wildcard symbol (*) was employed in the search. All search terms were searched using a multipurpose search (.mp) which retrieved articles with the search term in the abstract, heading word, title, original title, Medical Subline Headings (MeSH), and table of contents. The first stage was screening of articles based on the title and the abstracts. References of the articles identified in the search were examined.

### 2.2. Inclusion and Exclusion Criteria

Eligibility criteria for the studies involved several aspects. The study population included children, adolescents, and adults. Studies that reported mental health outcomes due to famine were included. The included studies were limited to countries in West Africa, only English articles, and original research utilizing either qualitative and/or quantitative data collection methods. Additionally, articles with full text available were included. Book chapters, opinion or commentary pieces, editorials, conference abstracts, and review protocols were not selected.

### 2.3. Data Screening and Extraction

EndNote X8 software was used to manage the reference library. After removal of duplicate references, all articles were exported to Covidence (Version 2.0), a software tool for screening of articles and data extraction for systematic reviews [29]. Study screening and data extraction were performed by two authors, WAD and JP, and one author, GD, resolved all conflicts. Included studies were extracted by WAD and JP using a data extraction sheet in Excel. The strengthening the reporting of observational studies in epidemiology (STROBE) statement provided guidance about the reporting of observational studies and facilitated critical appraisal and interpretation of the included studies [30]. Information extracted from included articles were consistent with the STROBE statement and included the aim of the study, outcome variables, study design and methodology, results, and general findings.

### 2.4. Quality Appraisal

The methodological quality assessment of all the papers was independently conducted by two reviewers before the papers were included for final review. The reviewers developed a critical appraisal check list using the JBI critical appraisal tools [31]. Study quality and strength of scientific evidence are seen as a vital component in systematic reviews [32]. In this narrative review, we used a six-point context for study designs that considers three categories: quality of reportage (three questions), decreasing risk of bias (two questions), and suitability of conclusions (one question). Quality and scoring discrepancies of reviewed papers were resolved through a discussion between two researchers. Of the seven papers included in this review, five were rated high quality and two papers had medium quality evaluation score. On average, the included studies scored high in the categories of quality of reporting and appropriateness of conclusions. The lowest scores and greatest variation were in the category of minimizing risk of bias. As most of the studies used a primary data source, the quality appraisal was very high in the description of the sampling frame on data source (see Table 1).

## 3. Results

### 3.1. Search Results

**Figure 1:**
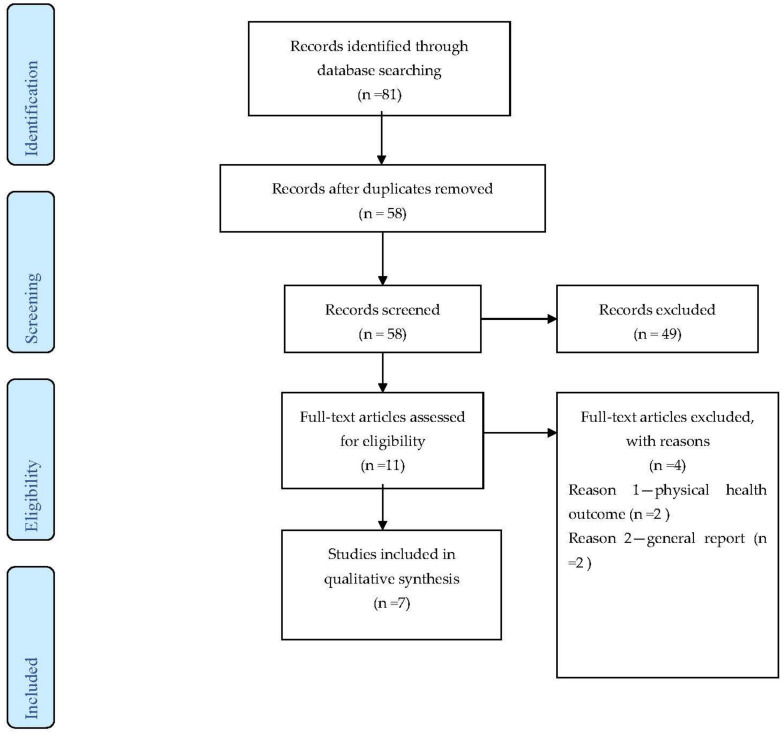
PRISMA flow diagram of the search and selection process.

A total record of (*n* = 81) articles was identified through journal database search. These articles were reduced to 58 after eliminating duplicate titles and screened for inclusion. Of these, 58 full-text documents were reviewed, and 48 records were excluded due to various reasons. During the full-text assessment for eligibility, eleven records were screened, and four studies were excluded for not having mental health outcomes related to famine and food insufficiency. Seven studies that met the inclusion criteria for this review were selected as illustrated in Figure 1. The Preferred Reporting Items for Systematic Reviews and Meta-Analyses (PRISMA) was used to show the number of records identified during the database search, included and excluded studies and the reasons for exclusions [33]. Due to the different types of studies included in this review, a meta-analysis was not performed.

### 3.2. Characteristics of the Literature

Five out of the six studies were based in Ghana. A variety of food insecurity impact on mental health articles were represented, and four of these papers were in rural and urban or suburban settings. Six studies collected primary data and only one study used experimental design (see Table 1).

From the included studies, food insecurity was measured based on the following: the number of months in the previous year that the respondent’s family reported being ‘unable to eat two square meals per day [34]; how often respondents went hungry because there was not enough food in their home in the past 30 days [35,36]; Household Food Insecurity Access Scale (HFIAS) [37]; and Household Hunger Score [38].

Mental health was measured with different instruments across the studies: Kessler non-specific psychological distress scale (K6) [34]; Kessler Psychological Distress Scale (K10) [35,36]; and the 17-item DUKE Health Profile, DUKE [37]. The K10 is an upgraded mental distress scales from the K6 scale that measures mental distress such as nervousness, hopelessness, restlessness or fidgety, worthlessness, tiredness, depressed, and sadness [34,39]. On the other hand, the 17-item DUKE Health Profile scale is a generic self-report that measures health status along four dimensions (anxiety, depression, pain, and disability) which are used to assess quality of life [40].

## 4. Discussion

Food insecurity has significant public health implications and has been widely recognized as such, globally [3,41]. However, in many countries this problem remains limited by a lack of population level data even among many high-income countries (HICs) [3,41]. Studies have reported higher prevalence of food insufficiency in LMICs compared to HICs and its independent association with a lifetime mental illness diagnosis [3]. Inadequate food intake has direct link to compromised nutritional status which negatively impact physiological mechanisms [3,4]. For instance, Dixon, Winkleby and Radimer [4] demonstrated that individuals from food-insufficient households had compromised physical health status compared to those from food-sufficient households. They also demonstrated that such compromised health status was linked to poor demonstrable physiological outcomes such as lower serum concentrations of critical nutrients which could predispose those individuals to chronic diseases. Additionally, they found that uncertainty about food availability affected the individual’s social and mental wellbeing by creating feelings of aggravation, worry and depression concerning food supplies [3]. Overall, the evidence from research conducted predominantly in HICs converge that food insecurity and insufficiency have negative health outcomes among individuals across the lifespan, both physical and mental health [2,3], and even worse among vulnerable population like women and children [2]. For instance, a study in Tanzania by Leyna, Mmbaga, Mnyika and Klepp [2] found that lower age, lower education, being a peasant, poor self-rated health status, and pre-existing health problems were linked to food insufficiency in women.

Most LMICs rely on aggregate data using food balance sheets and early warning systems that do not necessarily inform adequately on household-level food security and sufficiency [2]. This notwithstanding, the evidence from the studies reviewed showed that exposure to food insecurity is associated with increased psychological distress, worries and anxiety, sleep loss, intellectual disability, and general mental instability [34,35,36,37,42,43]. These findings are broadly consistent with literature from high-income countries [44]. The findings were grouped under the impact of famine on mental health and cognitive development. Strategies by stakeholders, including governments, to increase food production, prudent management of food stocks such as buying food early, reduction in the amount of food ration, and reduction in feeding frequency were proposed to improve food supply and mitigate the impact of food insufficiency among the population studied.

## 5. Impact of Food Insecurity and Insufficiency on Mental Health Outcomes

From the studies included in the review, food insecurity or insufficiency was linked to mental health problems and general wellbeing of individuals consistent with studies elsewhere, which have demonstrated that food insecurity is a significant factor associated with psychological distress among middle-aged and older adults [45]. One of the studies reported that moderate and severe food insecurity significantly increased psychological distress outcomes among both men and women, but this effect was greater with increase in age [36]. Another reported that mental health outcomes due to food insecurity were worse for females than males [37]. These findings are consistent with earlier studies in other LMICs such as South Africa, Botswana, Swaziland, and Ghana. Further, food insecurity and famine were associated with worries and anxiety, leading to weight and sleep disorders [43]. These findings are, again, consistent with earlier studies in other LMICs such as that in a study that found food insecurity provoked anxiety and stressful reactions among women in Tanzania [46]. However, the study by Sweetland et al. [34] did not find any associations between food insecurity and mental distress in Ghana or Nigeria. This finding contrasts with studies in the United States [47], Korea [48], and even in the other included studies in Ghana [35,37]. Overall, nearly all but one of the included studies were consistent with international literature.

Though within the scope of inclusion in this systematic narrative review, very scant evidence of published data is found in the West African sub-region. Other reports and studies from international organizations such as the World Health Organization (WHO) and the FAO confirm the findings of this narrative review. The WHO has reported that poverty increases risk for mental disorders and distress, and the associated disability may further worsen poverty in a vicious cycle [6], whereas positive mental health is associated with improved productivity and earnings, employment, educational achievement, health, and quality of life [49]. In support of this assertion, a review of studies conducted in low- and middle-income countries found positive associations between poverty and depressive, anxious, and somatoform symptoms [50].

## 6. Impact of Food Insecurity and Famine on Cognitive Function

Food insecurity, lack of access to food, and undernutrition have been linked with poor cognitive function [51]. Ampaabeng and Tan [42] found that childhood malnutrition negatively affected the cognitive development of 0–2 age group, similar findings were reported by Aurino et al. [38] among pre-school children. The studies also showed that the resultant effect of this decline in cognition persisted into adulthood [42]. Other studies have also reported a negative association between food insufficiency experienced in early or later life, and global cognitive function in middle-age and older adults [52]. Similarly, another study that examined food insufficiency and cognitive function in the elderly found that very low food security was prevalent among the elderly and was associated with lower cognitive performance [53]. The first five years of childhood is a critical stage for cognitive development and undernutrition at this stage is detrimental (Knudsen et al., 2006). Aurino et al. [38] reported that persistent food insecurity and undernutrition predicted decreased numeracy, literacy scores, short-term memory, and self-regulation compared with children from households without food insecurity. No significant gender differences were found between the included studies.

Ampaabeng and Tan [42] and Aurino et al. [38] studies were conducted in Ghana. The former used an existing dataset from the Ghana Education Impact Evaluation Survey (GEIES) in 2003, which measured intelligence based on Raven’s Progressive Matrices, as well as datasets from the Ghana Living Standards Survey II of 1988/89, the Demographic and Health Survey (DHS) of 1988, and the rainfall data from the World Bank’s Africa Rainfall and Temperature Evaluation System, to estimate the impact of food insufficiency and cognitive development. Aurino et al. [38], however, used a more recent longitudinal dataset (2015–2018) which was the Quality Preschool for Ghana project, an impact evaluation of a teacher in-service training and parental-awareness program in six districts in the Greater Accra Region. Both studies reported evidence of cognitive decline due to food insufficiency. Similar findings have been reported among immigrant children from food insecure households in the United States [54]. They found that immigrant children in food insecure households were worse in reading skills than nonimmigrant children in food secure households [54]. Moreover, food insecurity was associated with faster cognitive decline among Puerto Rican adults living in Boston in the United States [55]. Food insecurity experienced during the first five years of life is widely recognized as a key period of cognitive and physical development, during which the foundations for later cognitive and social functioning are laid [56].

## 7. Recommendations

Several recommendations have been made in the studies included in this review to address some of the challenges highlighted. First, there is the need for key stakeholders, such us governments, to initiate policies that would boost and attract people to invest in farming to improve food supply. This may be in the form of modernizing and mechanizing the agriculture sector, subsidizing farm inputs including seedlings, fertilizer, and machinery so that many people are able to access them for farming; creating an enabling environment for the youth to pursue farming and creating employment opportunities; and financing opportunities for agribusiness such as access to low interest rate and long-term repayment credit. Furthermore, encouraging West African governments to deliberately incentivize higher institutions of learning, such as the universities and research centres, to work collaboratively with farmers and agribusinesses to develop strategies for large-scale farming, and to address specific industry-related challenges. Additionally, construction of dams for dry-season farming will be another strategy to ensure all-year faming, especially in areas where there is only one rainy season and several months of dryness, and where people are unable to cultivate their farms. Additionally, advocacy and awareness programs about the links between food insecurity and poor mental health outcome should be funded and prioritized in policy initiatives by governments.

Due to the severe dearth of research on mental health and food insufficiency/insecurity in the West African sub-region, it is important to encourage more original research through appropriate funding supports by ECOWAS governments and their agencies as well as from international funding organizations. Such funding supports, in the view of the authors, are likely to stimulate more nuanced and sophisticated research and analyses to first, elucidate the complex and multifaceted issues around food security and mental health and second, provide adequate data for policy design and interventions. It is further recommended that conflict-sensitive policies, investments, and actions to reduce immediate food insecurity and malnutrition be implemented [1].

## 8. Limitations of the Review

This review has some limitations largely due to limited research in the area, language limitations, and scope resulting in very few published articles meeting the inclusion criteria. Future reviews should take into consideration the linguistic differences among countries in West African to capture other research work conducted in other official national languages such as French, German etc. Moreover, the limited number of studies makes it difficult to provide comprehensive and representative evidence of the impact of mental ill-health from famine or food insufficiency. Food insufficiency and insecurity was measured differently in all the studies, as was mental health. Regardless, the evidence strongly highlights the association between food insecurity/insufficiency and poor mental health outcomes. The review was only limited to studies conducted in English in West Africa and included studies from three countries—Ghana, Nigeria, and Burkina Faso—out of 17 countries in the West Africa region. Most of the studies in the West Africa Sub-region that reported famine and food insecurity did not measure mental health outcomes, hence their exclusion from this narrative review. This limits the generalizability of the review findings to only three West Africa countries. Again, limiting studies to only those published in English could have missed useful articles published in other languages.

In conclusion, food insecurity and insufficiency were closely associated with poor mental health and cognitive outcomes. Although there were limited studies in the sub-region, this study emphasizes the importance of further research to understand the mental health outcomes and the need for the development and implementation of comprehensive policies in addressing food production and its value chain.

## Figures and Tables

**Table 1:** Data extraction and quality assessment.

Author /Year	Study Country	Objective/Aim	Outcome Variables	Study Design	Target Population	Sample Size	Quality Appraisal	Key Results Summary
(Gyasi, Obeng & Yeboah, 2020)	Ghana	The effect of hunger on psychological distress in older age in Ghana. Whether the associations of hunger with psychological distress are differentiated by gender and age	Psychological distress	Cross-sectional	Older adults	1200	Medium	Food insecurity plays a critical role in psychological health and quality of life broadly of older people Exposure to moderate and severe food insecurity significantly increased the psychological distress score. No significant spatial variations exist in the association between food insecurity and psychological distress. The association is remarkable in men and older age group
(Ampaabeng & Tan, 2013)	Ghana	We examine the long-term effects of childhood malnutrition that was the consequence of a severe famine in 1983–84 in Ghana on cognitive development in adults 20 years later	Cognitive development	Experimental	Children	557	High	Differences in intelligence test scores can be robustly explained by the differential impact of the famine. Impacts are most severe for children under two years during the famine
Nanama & Frongillo (2012)	Burkina Faso	To understand household food insecurity by examining it in the context of subsistence farming, chronic food insecurity, and complex structure in northern Burkina Faso. To describe and analyze experiences of food insecurity and closely linked consequences at individual and household levels. To analyzed how these experiences influence household decision-making and priority-setting with regards to management of food insecurity	Mental health problems	Qualitative study	Adults	33	High	Food insecurity is closely linked with consequences such as concern, worries and anxiety that ultimately lead to weight and sleep loss. Food insecurity results in feelings of alienation (e.g., shame) and deprivation (e.g., guilt), and alters household cohesion leading to disputes and difficulties keeping children at home. Decisions made by household members to manage and cope with food insecurity are shaped by their fear of alienation and other cultural and social norms
Gyasi, Peprah & Appiah (2020)	Ghana	To examine how dietary patterns affect psychological disorders using data from adults 50 years and over	Psychological disorders	Cross-sectional	Older adults	1200	Medium	Moderate and severe food insecurity significantly increased PD score compared to no food insecurity PD score compared to no food insecurity. Having late daily meal was associated with increased risk of PD
Atuoye, & Luginaah 2017)	Ghana	To examine self-rated household food insecurity status and perceived mental health among household heads (both males and females) in Ghana, and indeed in SSA.	mental health elevated mental distress	Cross-sectional	Adults	1438	High	Poor mental health and related illnesses are becoming leading causes of morbidity in sub–Saharan Africa (SSA). Food insecurity is an important determinant of mental health among household heads. Compared to male household heads, females are more likely to report mental distress while food insecure. National food security strategy would improve food security and mental health in SSA.
Sweetland, Annika Claire et al. (2019)	Ghana and Nigeria	To examine the prevalence of and associations between food insecurity, mental distress and suicidal ideation in three rural village clusters in sub-Saharan Africa.	mental distress and suicidal ideation	Cross-sectional	Adults	762	High	High prevalence rates of moderate or severe mental distress in Nigeria and Ghana were higher than previously reported in the literature. Risk for suicidal was associated with food insecurity in Nigeria.
Aurino, E., Wolf, S., & Tsinigo, E. (2020)	Ghana	To investigate longitudinal associations between household food insecurity trajectories and multiple domains of early childhood development in lower primary school.	Cognitive development	Cross-sectional	Children	1333	High	Children from ever food insecure households had lower literacy, numeracy and short-term memory.

## Data Availability

The review used existing research data.
